# Comparative analysis of NAT reactivity and CLIA in detecting transfusion transmitted Infection among blood donors

**DOI:** 10.6026/973206300211947

**Published:** 2025-07-31

**Authors:** Jyoti Kala Bharati, Arvind Kumar Singh, Yatendra Mohan, Aaditya Shivhare, Shweta Chaudhary, Nouratan Singh

**Affiliations:** 1 Department of Transfusion Medicine, Uttar Pradesh University of Medical Sciences, Saifai, Etawah, Uttar Pradesh-206130, India

**Keywords:** Nucleic acid testing (NAT), Chemiluminescence immunoassay (CLIA), transfusion-transmitted infections (TTIs), blood donors, sensitivity, specificity

## Abstract

The effect of Nucleic Acid Testing (NAT) and Chemiluminescent Immunoassay (CLIA) in detecting TTIs (HIV, HBV, HCV, Syphilis and Malaria by rapid card) among 30,335 blood donations, with a focus on 1,843 reactive units is of interest. NAT showed superior sensitivity (98.50% for HBV, 98% for HIV and 97.50% for HCV) compared to CLIA (94.44.0% for HIV, 79.09% for HBV, 64.20% for HCV), but both methods exhibited high false-positive rates (37.7% for NAT, up to 70.6% for CLIA-HCV). NAT had specificity for HIV (98.5%), HBV (98%) and HCV (98%). CLIA exhibited high false positives (HBV: 27.1%, HCV: 16.5%, HIV: 5.7%), while NAT yield identified 106 HBV (0.35%) and 63 HCV (0.2%) additional cases. NAT was cost-effective for HBV and HCV but less so for HIV. Thus, NAT's role as a highly sensitive screening tool and with CLIA requiring confirmatory testing to optimize blood supply efficiency is shown.

## Background:

Transfusion-transmitted infections (TTIs), including human immunodeficiency virus (HIV), hepatitis B virus (HBV), hepatitis C virus (HCV) and Syphilis, remain a critical challenge in ensuring blood safety globally [1]. The risk of TTIs is particularly pronounced in high-prevalence settings, where undetected infections can compromise transfusion safety [2]. Chemiluminescence Immunoassay (CLIA) is widely used for its high sensitivity, rapid turnaround and cost-effectiveness [3, 4]. However, its limited specificity leads to false positives, necessitating confirmatory testing that increases costs and delays blood release [5, 6]. Nucleic Acid Testing (NAT) detects viral nucleic acids, offering higher specificity and the ability to identify infections during the window period, when serological tests may fail [7, 8]. NAT has significantly reduced residual TTI risk [9], but its high cost, specialized equipment and training requirements raise concerns about feasibility in tertiary healthcare settings [10, 11]. Recent studies highlight the complementary roles of CLIA and NAT in blood screening [12]. CLIA's high sensitivity makes it suitable for initial screening, while NAT's detection of low viral loads is critical for high-prevalence infections like HBV and HCV [13, 14]. The cost-effectiveness of NAT varies by TTI prevalence and healthcare infrastructure [15, 16]. Therefore, it is of interest to evaluate the comparative effectiveness of Nucleic Acid Testing (NAT) versus Chemiluminescence Immunoassay (CLIA) in detecting transfusion-transmitted infections (TTIs) among blood donors to optimize screening strategies.

## Methodology:

## Study design:

A retrospective cross-sectional study was conducted at a tertiary healthcare facility, analyzing blood donation records from January 2022 to December 2024. The study included 30,335 blood donations, of which 1,843 units were reactive for at least one TTI (HIV, HBV, HCV, Syphilis and Malaria) based on CLIA and NAT results.

## Data collection:

Blood samples were screened using CLIA (Abbott Architect i1000SR) for HIV, HBV, HCV and Syphilis and NAT ((Procleix Ultrio Elite Panther System, Grifols) for HIV, HBV and HCV. Malaria was tested using rapid cards. Data included blood type (ABORh), CLIA and NAT results, confirmatory outcomes and discordant results (CLIA+/NAT- and CLIA-/NAT+).

## Statistical analysis:

Sensitivity, specificity, PPV and NPV were calculated for CLIA and NAT using confirmatory tests as the gold standard. Chi-square tests assessed associations between CLIA, NAT and confirmatory results. McNemar tests evaluated discordances between CLIA and confirmatory tests or NAT. The Wilcoxon Signed Ranks test was used for HBV paired comparisons. NAT yield (CLIA-/NAT+ cases) was quantified to assess additional detection. Statistical significance was set at p<0.05. Analyses were performed using SPSS v25.

## Ethical considerations:

The study was approved by the Institutional Ethics Committee. As a retrospective analysis, informed consent was waived. Data were anonymized to ensure confidentiality.

## Results:

In this study of 30,335 blood donations, 1,843 units, representing 6.08% of the total, tested reactive for at least one transfusion-transmissible infection (TTI). The distribution of blood types showed B Positive (28.78%), O Positive (28.15%) and A Positive (25.54%) as the most common, with O Negative (9.41%) and AB Negative (7.2%) having the highest reactivity rates. Among reactive units, HBV was the most prevalent TTI (23.6%, 435/1,843), followed by Syphilis (17.0%, 313/1,843, CLIA-based), HCV (5.5%, 101/1,843) and HIV (0.8%, 14/1,843). Co-infections included HBV & HCV (3.8%, 70/1,843), HIV & HCV (0.2%, 4/1,843), HBV & HIV (0.1%, 1/1,843) and HBV, HCV & HIV (0.1%, 1/1,843). Relative to total donations, TTI prevalence was low: HBV (1.4%), Syphilis (1.0%), HCV (0.3%) and HIV (0.05%) had shown in Table 1 (see PDF) and figure 1 (see PDF). CLIA exhibited higher reactivity rates than NAT for all infections: HIV (0.4%, 122/1,843 vs. 0.06%, 18/1,843), HBV (3.0%, 904/1,843 vs. 1.7%, 507/1,843) and HCV (1.4%, 421/1,843 vs. 0.6%, 176/1,843) of total donations, indicating a higher false positive rate shown in Table 2 (see PDF) and Figure 2 (see PDF). Syphilis CLIA reactivity was 1.0% (313/1,843) and Malaria was detected in 0.003% (1/30,335) of donations. Overall, NAT reactivity was 3.3% (994/30,335) of total donations.

Sensitivity and specificity analyses revealed distinct performance profiles for CLIA and NAT shown in Table 3 (see PDF), Figure 3a, b (see PDF). For HIV, CLIA showed 94.44% sensitivity and 94.25% specificity, with 13.93% PPV and 99.94% NPV, indicating reliable negative results but many false positives due to low PPV. NAT showed 98.00% sensitivity and 98.50% specificity, with 79.03% PPV and 99.88% NPV, offering higher accuracy for detecting and confirming cases. For HBV, CLIA showed 79.09% sensitivity and 62.57% specificity, with 44.51% PPV and 88.75% NPV, reflecting moderate performance with notable false positives and negatives. NAT showed 98.50% sensitivity and 98.00% specificity, with 94.87% PPV and 99.39% NPV, demonstrating high reliability. For HCV, CLIA showed 64.20% sensitivity and 84.13% specificity, with 29.35% PPV and 95.81% NPV, indicating poor detection of true cases and many false positives. NAT showed 97.50% sensitivity and 98.00% specificity, with 83.90% PPV and 99.76% NPV, performing strongly. NAT consistently outperformed CLIA across all infections, particularly in PPV, making it ideal for confirmation, while CLIA's high NPV supports its use for initial screening but requires NAT follow-up due to lower accuracy. Discordant Results and NAT Yield highlighted differences between CLIA and NAT shown in Table 4 (see PDF) and Figure 4 (see PDF). CLIA+/NAT- rates were high for HBV (1.6%, 500/1,843), HCV (0.9%, 304/1,843) and moderate for HIV (0.35%, 105/1,843), confirming CLIA's false positive tendency. NAT yield (CLIA-/NAT+) was significant for HBV (0.35%, 106/1,843) and HCV (0.2%, 63/1,843), but minimal for HIV (<0.01%, 1/1,843), demonstrating NAT's role in detecting cases missed by CLIA. Statistical Associations confirmed significant associations and discordances shown in Table 5 (see PDF). Chi-square tests showed strong associations between CLIA and confirmatory results for HIV (χ^2^=385.448, p<0.001), HBV (χ^2^=255.364, p<0.001) and HCV (χ^2^=220.905, p<0.001) and between NAT and NAT_CONFIRM (χ^2^=771.017, p<0.001). McNemar tests indicated significant discordances for HIV (χ^2^=85.263, p<0.001), HBV (χ^2^=254.866, p<0.001) and HCV (χ^2^=129.146, p<0.001), with CLIA overestimating positives. The Wilcoxon Signed Ranks test for HBV paired comparisons was significant (Z=-16.005, p<0.001). Cost-Effectiveness and Feasibility analysis indicated NAT's high yield for HBV (106 cases) and HCV (63 cases, total 0.56% of donations) justified its use in high-prevalence settings (HBV: 1.4%, HCV: 0.3%). HIV, low prevalence (0.05%) and minimal yield (<0.01%) suggest limited cost-effectiveness. Feasibility in tertiary settings is constrained by NAT's high costs and infrastructure needs, supporting a tiered CLIA-NAT approach.

## Discussion:

This study confirms NAT's higher sensitivity for HBV (98.5%), HIV (98%) and HCV (97.5%) compared to CLIA (HIV: 94.44%, HBV: 79.09%, HCV: 64.2%), consistent with evidence that NAT reduces the window period for viral detection [1, 7, 17]. NAT's ability to detect low viral loads is critical for HBV and HCV, which showed higher prevalence (1.4% and 0.3% of total donations, respectively) [13, 18]. In contrast, NAT's sensitivity for HIV (98%) compared to CLIA (94.44%) suggests NAT's adequacy for HIV screening in low-prevalence settings (0.05%), as supported by recent studies [2, 12, 14, 19]. CLIA's high false positive rates (HBV: 27.1%, HCV: 16.5%, HIV: 5.7%) align with reports of serological assay limitations, necessitating confirmatory testing to avoid donor deferral [3, 5, 6, 20]. NAT's yield (HBV: 0.35%, HCV: 0.2% and HIV: <0.1%) enhances blood safety, particularly for HBV and HCV, corroborating findings from high-prevalence regions [4, 8, 21]. The cost-effectiveness of NAT is evident for HBV and HCV, where high prevalence and yield justify resource allocation [15, 22, 23]. HIV, the minimal yield (<0.01%) supports selective NAT use, as CLIA's high sensitivity minimizes residual risk [16, 24]. Feasibility in tertiary settings is constrained by NAT's high costs, specialized equipment and training requirements, consistent with global challenges [10, 11, 25]. A tiered CLIA-NAT approach optimizes safety and resource use, as advocated in recent guidelines [12, 26, 27]. Future studies should explore automated NAT platforms to reduce costs.

## Conclusion:

NAT significantly enhances blood safety by detecting HBV and HCV cases that are missed by CLIA. However, CLIA is adequate for HIV screening. Thus, a tiered CLIA-NAT strategy optimizes safety and resource use in tertiary settings. Future studies should evaluate Syphilis confirmatory testing and cost-benefit analyses to refine screening protocols.
